# “Don't Promise Something You can't Deliver:” Caregivers' Advice for Improving Services to Adolescents and Young Adults with Autism

**DOI:** 10.1155/2023/6597554

**Published:** 2023-03-21

**Authors:** Kristen A. Berg, Karen J. Ishler, Sarah Lytle, Ronna Kaplan, Fei Wang, Tugba Olgac, Stacy Miner, Marjorie N. Edguer, David E. Biegel

**Affiliations:** ^1^Jack, Joseph and Morton Mandel School of Applied Social Sciences, Case Western Reserve University, 10900 Euclid Ave, Cleveland, OH 44106, USA; ^2^Center for Health Care Research and Policy, The MetroHealth System, 2500 MetroHealth Dr, Cleveland, OH 44109, USA; ^3^University Hospitals Cleveland Medical Center, 11100 Euclid Ave, Cleveland, OH 44106, USA; ^4^Cleveland State University, College of Health, 2121 Euclid Ave, Cleveland, OH 44115, USA; ^5^Frances Payne Bolton School of Nursing, Case Western Reserve University, 10900 Euclid Ave, Cleveland, OH 44106, USA

## Abstract

Approximately 50,000 youths with autism spectrum disorders (ASD) exit U.S. high schools yearly to enter adult systems of care, many of whom remain dependent on family for day-to-day care and service system navigation. As part of a larger study, 174 family caregivers for adolescents or young adults with ASD were asked what advice they would give service providers about how to improve services for youth with ASD. Reflexive thematic analysis identified a framework of five directives: (1) provide a roadmap to services; (2) improve service access; (3) fill gaps to address unmet needs; (4) educate themselves, their families, and society about autism; and (5) operate from a relationship-building paradigm with families. Education, health, and social service providers, as well as policymakers, can use these directives to better assist youth with ASD and their families in the transition to adulthood.

## 1. Introduction

Approximately 50,000 youths with autism spectrum disorders (ASD) leave high school each year in the U.S. [1], equating to roughly half a million young people with ASD entering adulthood over the next decade [1]. ASD is deemed a lifelong developmental disorder, and, while often diagnosed in childhood, many individuals with ASD retain significant difficulties in adaptive functioning and social roles throughout life [2]. Consequently, individuals often remain dependent on family caregivers to ensure care through adulthood [3, 4]. Of specific concern to families are problems encountered as youth age out of the school system and attempt to access adult systems of care. The transition out of high school is often accompanied by a loss of service access and limited employment opportunities [5–7]. Despite their crucial role in interfacing with service providers, research has scarcely explored caregivers' voices regarding how service landscapes should best serve transition-age adolescents and young adults with ASD.

Ensuring care for older ASD youth may be difficult due to service system barriers, including waiting lists for Medicaid waiver care [8, 9], ASD stigma among healthcare providers [10], out-of-pocket expenses for supportive services [11], and opportunity costs related to caregivers' service coordination [12]. Although multiple challenges exist in ensuring care for older youth with ASD, most research has focused on young children and their caregivers. To date, few studies exist on older youth, and even fewer have featured their caregivers' voices. Parents report feeling as if they are “falling off a cliff,” referring to the dramatic drop in service availability after high school exit [1, p. 25], and research broadly documents a high level of unmet service needs for older and transition-age youth compared to younger children [7, 13].

Existing qualitative evidence on the transition to adulthood has explored parents' and youths' perspectives on the transition process [14] and what constitutes desirable outcomes [15]. Research has also examined caregiver and youth perceptions of barriers to and facilitators of success in terms of employment and postsecondary adjustment [16, 17]. Additional work has integrated caregivers' voices into the discussion of unmet youth service needs [18]. Caregiver feedback has been used to evaluate specific ASD programs and services, including Medicaid-funded ASD service programs in Pennsylvania [19] and a social skills-focused vocational training program in Alabama [20]. Caregiver perspectives have also been featured in studies of recreational activities [21] and primary care services for transition-age youth with ASD [22]. Yet, caregivers have not been asked directly for advice about how to improve services generally for transition-age youth. Because family caregivers must often schedule and coordinate services [1], they offer an important perspective on the services received by ASD youth.

Our work critically expands prior research by explicitly seeking caregivers' advice from service providers for improving care for transition-age youth. In contrast to existing work, our study did not direct caregivers to consider a specific service or service system. Rather, the current study's purpose was to amplify caregivers' own priorities in recommending improvements to system-level services and supports provided to transition-age youth with ASD. Below, we follow Tong et al.'s [23] established COREQ guidelines for reporting on qualitative research.

## 2. Methods

### 2.1. Study Overview and Recruitment

This qualitative investigation is part of a larger study of service use and well-being among transition-age adolescents and young adults with ASD and their family caregivers in Northeast Ohio [24]. Family caregivers across 14 counties in Northeast Ohio were recruited from sources including: ASD community-, hospital-, and school-based programs; county developmental disability boards; advocacy groups; religious institutions; and health and human service agencies. Study flyers were distributed via organization websites and social media and at local education and fundraising events. Caregivers were referred by 28 different agencies and organizations.

Eligibility requirements included the following: (1) being the primary caregiver; (2) to an adolescent or young adult (age 16 to 30); (3) who had been diagnosed by an education or health professional with an ASD (e.g., autism, Asperger's, PDD-NOS). The lower age of 16 was selected to be consistent with the age at which transition plans are required to be written into individual educational plans. A generous upper age limit of 30 was adopted in response to three factors: (1) the paucity of research on the experiences of ASD youth as they approach and exit high school; (2) laws in several states entitling individuals with disabilities to remain in school into their early 20s; and (3) evidence suggesting lags in service access that can result from Medicaid eligibility reevaluation at age 18 or the loss of health insurance coverage under a parent's policy at age 26. No exclusion criteria (e.g., having comorbid disorders, the caregiver not co-residing with the youth) were specified. Semistructured interviews were conducted over 18 months, between May 2017 and November 2018, by trained graduate-level research assistants at caregiver-designated locations. The study was approved by the Institutional Review Board at Case Western Reserve University. Caregivers received a $25 gift card for participation.

### 2.2. Study Sample

A total of 239 caregivers were referred and screened for the study; 31 were ineligible based on the age of the youth with ASD, and 34 could not be reached, were not scheduled, or ultimately chose not to participate. Completed interviews were conducted with 174 caregivers (83.7% of those eligible). Caregivers were primarily mothers (91.4%), with a mean age of 54 years, and were caring for primarily male (72%) youth with ASD who were 21 years old on average. Caregivers were fairly well educated, and most were employed and married or partnered. Nearly one-fifth reported income below $40,000. Descriptive data regarding caregivers and youth with ASD are presented in Table 1. Details about the measures used to collect these data are described elsewhere [24].

Over 40% of youth were enrolled in secondary or high school; less than 13% were attending college or postsecondary vocational training. Of those no longer in school, 60.5% were currently working, and most (73.5%) were working only part-time. Nearly 45% required at least consistent support with daily activities and supervision much of the day across all settings (e.g., home, school). The most commonly utilized services caregivers reported youth utilizing were medical (66%), mental health (63%), employment supports (54%), case management (53%), educational (51%), social supports (46%), and life skills (45%).

### 2.3. Procedures

Data were drawn from two open-ended questions that were embedded in a broader interview composed mainly of standardized measures. The primary question focused upon in this study was asked in a section on service use and experiences. Interviewers asked, “What advice would you give to service providers about how to improve services for adolescents and adults (like [youth's name])?” Almost all (*n* = 166, 95.4%) provided responses, which were then analyzed; eight respondents indicated having no advice to give. Secondarily, the interview's closing question asked if there was anything else the caregiver wanted researchers “to know about you and your family's needs or situation in providing care to (youth's name).” Over 40% offered a closing comment that elaborated on or extended their earlier response; these comments were also analyzed. The word counts of these qualitative responses were 31 on average, ranging from 6 to 332. Interviews lasted between 50 and 180 minutes, largely owing to the use of standardized scales, with an average length of 95 minutes.

Graduate-level research assistants were trained to interview and reconstruct the thematic content and dialogue of participants' extended responses and to record salient phrases (see [26]) verbatim. All interviewers were trained to probe for elaboration and clarification using Gorden's [27] verbal (e.g., “Could you spell that out a little more for me?” or “When you say [keyword], could you help me understand more what you mean?”) and nonverbal strategies (e.g., utilizing active silence). Interviews were not audio-recorded. This decision is supported by methodological research showing that written field notes from carefully trained interviewers yield levels of detail and thematic accuracy equivalent to those of transcripts from audio-recorded interviews [28]. Interview documents were reviewed by project staff within 48 hours, and interviewers were asked to explain any unclear entries; on occasion, caregivers were recontacted to clarify responses.

#### 2.3.1. Data Coding and Analysis

We employed inductive reflexive thematic analysis [29], guided by Miles and Huberman's [30] framework, with data abstraction and configuration proceeding iteratively. After data collection ended, data reduction was initiated wherein all coauthors studied caregivers' verbatim responses and generated a list of first-pass, data-driven codes. That initial list was transferred to an Excel spreadsheet, where three coauthors (Authors 1, 3, and 4) independently applied the initial codes to each response. If needed, new codes were created to comprehensively abstract the data. A fourth researcher (Author 2) reconciled differences in coding. Author 1 is a rehabilitation counselor and experienced qualitative researcher; Author 2 is a social worker; Author 3 is a music therapist; and Author 4 is a pediatric psychiatrist. All authors have experience working with individuals with developmental and/or intellectual disabilities.

Data were then transferred into NVivo version 12.6.0 [31] to facilitate data organization and configuration. Data display transpired when coders organized data-driven codes into thematic categories using pile-sort techniques [32]. This generated a combination of semantic and latent subthemes through which higher-order themes and their patterns of interrelationship were defined. Interpretive insights from the pile-sort process, as data were compared back against themes and subthemes, were tracked by memoing. Coded data were compared iteratively until no new concepts were identified in relation to the study's core research aim [33, 34].

#### 2.3.2. Conclusion-Drawing and Verification

Data verification and trustworthiness were enhanced through various mechanisms. We made efforts to reflexively engage with data and analysis by (a) continually bracketing, through memos, coders' existing assumptions about service quality for families and youth with ASD in light of first-pass coding and (b) continually reexamining those earlier existing assumptions against later and final coding, analysis, and interpretation of findings [35]. Member checking was employed when key findings were presented to an audience of roughly 100 professionals and family members at a regional ASD conference. Subsequent, invitation-only workshops engaged attendees in facilitated discussion around the study's findings. Nineteen family caregivers who participated in the study and attended one of the workshops confirmed that the identified themes were consistent with their experiences, supporting the credibility of our thematic reconstruction and the reliability of our field note strategy for documenting participants' responses.

## 3. Findings

**Figure 1:**
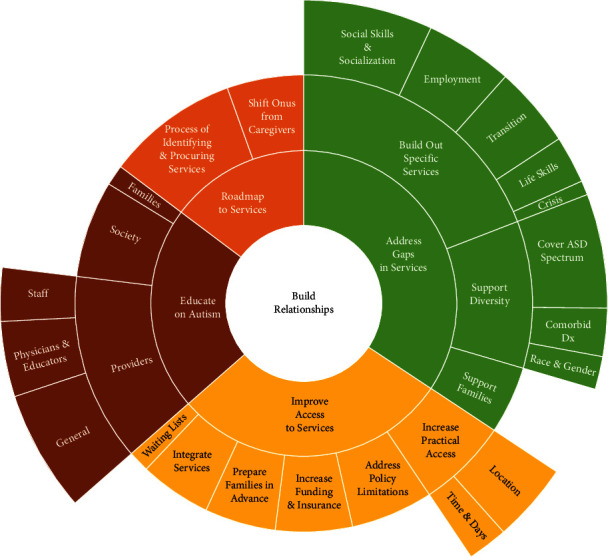
Findings from reflexive thematic analysis of caregivers' advice to service providers. Note. ASD: autism spectrum disorder; Dx: diagnoses; Tx: therapies.

The analysis identified five primary themes that consist of both a logistical (four themes) and relational (one theme) framework of recommendations for how service providers could improve services for youth with ASD. These comprise the two innermost concentric circles in the sunburst diagram in Figure 1. Four logistical directives implored providers to (1) equip caregivers with a clear roadmap to the service landscape (*n* = 48); (2) improve access to services by reducing barriers (*n* = 129); (3) fill service gaps to address unmet needs (*n* = 129); and (4) better educate themselves, other professionals, families, and society at large (*n* = 60). Finally, caregivers contended (5) that providers must operate from a relationship-building paradigm with youth and families (*n* = 77).

The second innermost concentric circle displays caregivers' four core logistical directives (roadmap to services, improve access to services, address gaps in services, and educate on autism), with subthemes emanating out through sun rays comprising the two outermost rings. Sun ray width corresponds to the frequency with which caregivers mentioned a particular subtheme. The center circle conveys advice from caregivers that the four logistical directives be enacted from a place of relationship-building with youth with ASD and their families.

### 3.1. Roadmap to Services

Caregivers described the need for a clear “roadmap” (metaphor invoked by caregivers during member checking) for navigating the service landscape. Two components comprised the roadmap.

#### 3.1.1. Clarify Process of Identifying and Procuring Services

First, caregivers needed clarification regarding the process of identifying and procuring available services, stating, “Providers need something clear and concise for what's available for families and steps to take.” Another reflected on the inadequate counsel received when attempting to connect with necessary services: “Once (son) was diagnosed, I wasn't given enough guidance in getting connected with services and information on what to do.” One mother's current uncertainty communicated lack of process improvement over time: “We are not getting many services. I'm not sure I'm looking in the right direction or contacting the right people.” Some identified settings through which information about services might best reach families. For example, referring to medical care, one commented, “It is really important for families to have information on services available and for physicians to better guide parents.” Another identified the school system as a critical point of contact: “(There) should be more information when they are in school. (We were) not notified of what services are available.”

#### 3.1.2. Shift Onus from Caregivers

Beyond clarifying processes for securing services, caregivers advised that providers shift the excessive onus of locating and coordinating services away from caregivers, reflecting, “So much falls on me as a parent” and “It would've been nice if services could have come to me instead of me searching for services.” Others articulated the burden of service location, instructing, “Let families know what is out there… It's stressful enough raising the child without having to research what I need for transitions.” Procuring services was a common struggle: “We struggle and fight… I have to fight for every service. The answer is always ‘no' in pursuit of services until I have to kick someone's ass.”

### 3.2. Improve Access to Services

Following critical steps of roadmapping, caregivers advised providers to measurably improve access to existing services, commenting on barriers (e.g., inadequate funding, policy limitations, extensive waiting lists). Caregivers suggested several changes that would increase access to services in a practical way.

#### 3.2.1. Integrate Services

Caregivers reflected on the value of a more integrated service system, stating, “It would be great if they could have a one-stop shop for getting all services” and “There needs to be a place that lets you know everything available.” Others alluded to the education system as a key touchpoint for gathering critical information, advising that “High schools need to be better educated on services out there to help caregivers.”

#### 3.2.2. Prepare Families in Advance

Caregivers underscored the importance of providers helping to prepare youth with ASD and their families well before key transitions. One mother simply advised, “Get to the pipeline early.” Caregivers alluded to shortcomings of school systems to help prepare for transition to adulthood, instructing “Educate parents early on about transitioning from childhood to adulthood to make the transition smoother.” For another, vocational assessment did not begin soon enough: “There was lots of testing after high school with nothing in place. (Son) got used to not doing anything. I would like to see testing start earlier and programs put in place for graduation.”

#### 3.2.3. Increase Funding and Insurance Coverage

Numerous caregivers noted the need for more funding, and funding that could more efficiently address youths' needs. Concerned about cost, one mother mentioned the need for flexible fee structures: “It's a very expensive condition. Having services that have sliding scales for those in need is important.” Another observed the sometimes mutual exclusivity of affordable and high-caliber services, asserting, “There's the financial part that can be hard; good providers often don't take Medicaid.” Limitations of private insurance were noted: “Insurance needs to cover more mental health services.”

#### 3.2.4. Address Policy Limitations

For some, problems with service access were symptoms of policy limitations. A father expressed frustration with restrictive policies for accessing certain types of housing:

“Our federal government has taken mandate over waivers, and they don't understand what families or individuals need. They dictate the types of environments they think individuals should live in and are making those environments more isolated than inclusive.”

#### 3.2.5. Reduce Waiting Lists

Related to service system shortcomings, several caregivers noted an extensive wait time for services: “Waiting time for services is too long. There needs to be better access.” Caregivers also expressed concerns about waiting lists for home- and community-based waiver services, declaring, “The waiver list is too long. We shouldn't have to wait 19 years!”

#### 3.2.6. Increase Practical Access

Caregivers further advised providers to offer greater flexibility in the locations, times, and days of service provision. One mother explained, “One size does not fit all by any stretch of the imagination. Services need to be available and not just during the day.” Another caregiver noted scheduling discordance for those working traditional hours: “Some families need creative scheduling because some parents work 9:00 a.m. to 5:00 p.m.” Others suggested that providers could better accommodate the unique circumstances and needs of youth, stating, “Meet somewhere else if transportation is a problem” and “Home visits would be helpful because he's more comfortable there.” Other caregivers noted geographic imbalances in services, voicing, “We need more services and support groups in the inner city.”

### 3.3. Address Gaps in Services

In addition to improving access to services that caregivers identified as existing but unreachable due to myriad barriers, caregivers also identified the absence of needed services and the absence of appropriate training for professionals.

#### 3.3.1. Build Specific Services

Caregivers perceived a general dearth of services and identified specific ones as lacking. Requests for social skills training and socialization services were common, with one mother suggesting, “Offer peer social activities. He needs caregivers outside of the family or day program to engage him in social activities.” Caregivers also advised providers to focus on “daily living skills” and “basic life skills” that would transfer to “real life.”

Caregivers suggested providers increase employment services. One mother alluded to the inadequacy of the assistance her son received from a vocational rehabilitation agency, asserting, “Kids like (son) need more work experiences. The vocational agency only gave him one work opportunity, but kids need more.” Another commented on the scarcity of vocational support for youth with ASD and the lack of opportunity to engage with society:

“There is a general lack of employment services for people with autism in competitive employment. It's a struggle to find providers to work with. I know many children who are just sitting there and could have contributed to society.”

Caregivers highlighted the need for improved transition services, noting, “This transition to adulthood is scary… There need to be solutions for children past 18 and 26 (years old),” and “Transitioning out of high school is poorly done. We don't have enough information for the next phase.” About the dearth of information, another caregiver explained, “My district does not have a transition coordinator. I don't know who to talk with other than the guidance counselor, who has not been very helpful.” Others identified an urgent need for residential crisis stabilization:

“(I) wish there were places to take them where they can stay and be safe until they calm down, to help parents deal with anger and outbursts when kids get out of control. (We) can't go to the hospital because if they're not an immediate threat, the hospital won't keep them. The police can't help unless they actually hurt someone, and sometimes cops make things worse.”

#### 3.3.2. Support Diversity

Caregivers of youth across diverse points on the spectrum voiced frustration at their exclusion from services based on level of functioning. Some who described their youth as “high-functioning” asserted that such functioning thwarted service reception. For example, one mother explained:

“A better understanding of needs is important for someone who's high-functioning; (daughter) often doesn't receive services because she doesn't appear to need them when compared to individuals with more severe symptoms.”

However, caregivers of youth they described as “low-functioning” expressed a parallel sentiment: “There are many services for younger kids, newly diagnosed individuals, and individuals with higher functioning. My daughter is low-functioning, nonverbal, and needs one-on-one support.” Another noted, “Some programs only serve kids with a certain IQ. Don't call yourself an 'autism program' if you only serve kids with Asperger's.”

Several caregivers identified support needs for ASD youth's intersecting identities. Alluding to the minority of girls with ASD, one mother advised, “Take into consideration that there are girls on the spectrum” by offering diverse socialization activities. Others predicted a growing need for providers' understanding of how ASD intersects with gender identity. One grandmother shared that her loved one “…can't find someone who knows autism and transgender issues.” Additional caregivers articulated concerns about comorbid mental health conditions, stating that “… autism and mental illness interactions are often not talked about.”

Some caregivers identified a lack of socially inclusive services for persons of color. One mother distilled that “People who are of color and have autism, and their families, struggle and feel excluded.” Another echoed such concerns: “In the Black community, there is a lack of understanding and support of those with Asperger's or autism. This needs to improve.”

#### 3.3.3. Support Families

Caregivers emphasized the need for services that support family functioning. The stress of rearing a child, and then adult, with ASD profoundly affected some families and marriages. A mother advised providers to infuse support for family relationships early on in the caregiving trajectory: “Caring for (son) caused the divorce; it really fractured my family. There needs to be marital therapy when a child is diagnosed. Even support for other children.” In contrast, a father reported that his son's ASD had promoted family closeness. Yet, he also advised attention to siblings' support needs, stating, “(Son) keeps us together as a family; we've grown closer. Address issues of sibling support; my kids sacrifice a lot having an older sibling with severe special needs… (it is) not a typical teenager's life.” Another caregiver commented on scarce support for neurotypical siblings: “(My) younger child has had difficulty adjusting to life with an older sibling with autism… feels embarrassed by his actions… I wish there was support for siblings to learn strategies about how to manage frustrations.”

### 3.4. Educate on Autism

#### 3.4.1. Educate Providers

Caregivers advised providers to educate themselves and other providers about ASD, focusing on “Training of staff… staff needs to understand autism” and generally instructing “Stay current with your professions.” Another detailed that “School does great. Others, like health aides, need training on how to better care for someone with autism.” Educators were also perceived as needing better training on ASD. Caregivers stated, “Teachers need to be more knowledgeable about how to relate to (son)” and “Educators should know more about autism.” Others noted the need for better-educated physicians, from pediatricians (“There needs to be better education for pediatricians for directing parents with newly-diagnosed children; we need to know what to expect”) to general practitioners caring for youth as they move into adulthood (“As a large number of adolescents with autism enter adulthood, physicians have to learn to deal with them”).

#### 3.4.2. Educate Families

Several caregivers identified a need for more family education. One simply instructed, “Make the parent feel like they can handle a child with severe autism.” Another asserted, “More caregivers need to be educated about their family members in order to provide better-quality support and get services.”

#### 3.4.3. Educate Society

Caregivers underscored the need for societal awareness and education, alluding to experiences of intolerance and disrespect. They urged providers to “…educate the public on autism; educate adults on respect” and identified that “the main problem we encounter is an intolerant society.” A mother shared how lack of awareness contributes to family isolation:

“I don't think other people understand how much it affects your life. We try to appear normal, but getting it out is so hard. Going out into crowds is hard. It amazes me how narrow-minded people can be, thinking that you should keep your child at home or that their kids will be scared.”

A Black mother articulated the acute danger and fear of her son's victimization by the police: “(We are) fearful that if a cop asked (son) to stop, the worst outcome would result, like him getting shot. (It is) difficult getting (son) to services such as the barbershop. The community, especially the inner city, needs to be more educated.”

Underscoring that all individuals, regardless of ability, share identical needs for respect and dignity, one mother said, “They are the same as you and I. The world has a long way to go.”

### 3.5. Build Relationships

Nearly half of the caregivers (46%) who provided a logistical directive for how services could be improved also articulated a corresponding need related to relationship-building.

#### 3.5.1. Person-Centered Care

Caregivers directed providers to operate within person-centered frameworks of care, advising, “Providers must get to know individual patients” and “Definitely listen to an individual's specific needs.” One mother noted that person-centered approaches and their variants might still be considered non-traditional among providers: “Think outside of the box: (utilize) trauma-informed care, person-centered care.” Another elaborated, “Focus on (youth) being people first, the person and not the disability. But understand the disability.”

#### 3.5.2. Qualities of Successful Providers

Compassion, empathy, and patience were identified as foundational qualities of successful providers. Caregivers directed, “Be patient, use repetition, and stay calm,” and “Listen to the person with autism. Talk to them as a person. Have patience. Know who you are talking to, and adjust your interaction accordingly.” Another underscored the need for empathy and curiosity in attempting to improve services for youth:

“Too often, there are assumptions and judgments being made about why individuals with autism do what they do. Try to understand the behavior first. Have empathy for what it's like to feel overwhelmed and live in a world that makes no sense. Stop looking at the behavior first. Seek to understand.”

#### 3.5.3. Strategies to Build Relationships

Caregivers offered key relationship-building strategies for providers. They underscored attunement, directing providers to listen (“Really listen to the person with autism”), engage (“Engage him; he only has behavior problems when bored”), focus on strengths (“Focus on strengths, minimize difficulties and problem behaviors”), and withhold damaging assumptions (“Do not assume they are unintelligent”; “Control your prejudice. Children with autism are not ‘lazy'.”).

Caregivers implored perspective-taking, reminding providers that ASD youth “…will not respond like a typical person will; try to understand their perspectives,” and instructing, “Put yourself in their place. Treat them as an adult and with respect. Give them responsibility; let them have an active role in their lives.” Caregivers urged providers to “spend more time with families” to better understand their worlds, advising, “Take more time in getting to know the family. Sometimes I feel like I'm just a number.”

One final strategy for relationship-building was to collaborate with families. One caregiver instructed, “Listen to the parents and work with parents who have a lot of experience.” Others identified clear, trustworthy communication as key, highlighting transparency and follow-through as indispensable: “They must do what they say they'll do. Be transparent about training and responsibilities…Develop a relationship, based on trust, with families.” Multiple caregivers reiterated these notions. One caregiver implored:

“Don't promise something you can't deliver. Over the years, you work with people who say they'll provide something, and then, either because they lack the skills or have made unrealistic promises, they don't deliver.”

## 4. Discussion

The current study synthesized advice from caregivers into five key recommendations for improving services for youth with ASD. Caregivers called on providers to: (1) provide a road map for navigating the service landscape; (2) improve access to services; (3) address substantive gaps in services; and (4) educate themselves, other professionals, families, and society about ASD. They also advised providers to (5) adopt relationship-building approaches with both youth and their families. We discuss below the implications for practice, policy, and future inquiry.

### 4.1. Roadmap

Our finding that caregivers lack a clear “roadmap” of services converges with a recent U.K. study in which fathers noted the lack of a “route map” for procuring support services for youth with ASD [36]. These findings bolster existing quantitative evidence that information about services is a principal unmet need of family caregivers [37, 38]. Caregivers in this study reported having to shoulder inordinate responsibility for locating and coordinating services. Similar sentiments have been reported by parents of younger children with ASD; for example, caregivers in the Netherlands experience “overburdening” [39], and parents in British Columbia describe service coordination as a “full-time unpaid job” [40]. It is concerning to hear these themes reflected in the comments of caregivers to transition-age youth. Mollifying strains of resource coordination should be a priority for policymakers, researchers, and providers, as prior research has established that such strains are associated with ASD caregivers' poor emotional outcomes [41]. Efforts to shift the onus of service-seeking and coordination from caregivers could provide meaningful support to caregivers. Improved models of ASD care might centralize service provision into “one-stop-shop” centers, such as the Ohio State University's Nisonger Center [42] and other University Centers for Excellence in Developmental Disabilities, that integrate specialized therapies and clinical care for youth as well as training and counseling for other family members.

### 4.2. Access to Services

Study findings regarding difficulties in accessing services are consistent with prior quantitative and qualitative research. In the U.S., difficulties have been reported in national studies [1, 38], as well as studies confined to the Midwest [43, 44], Southeast [45], and Mid-Atlantic regions [18, 19]. On some indicators, ASD youth appear to have similar or even greater access to care than neurotypical youth; for example, a greater percentage are covered by insurance and have a usual source of care [46]. However, youth with ASD still experience considerable barriers (e.g., long waiting lists), resulting in delayed and unmet care needs [47]. Recent work suggests the benefits of training caregivers to advocate for adult services [48].

Access-related barriers do not appear unique to a specific type of service; difficulties have been reported in accessing healthcare [46], employment [15], and recreation programs [21]. Some past work has focused on the need to help youth with ASD transition to adult healthcare and mental health services (e.g., [49]). Although it is important that individuals receive services from appropriately trained providers, the current study provides support for a “medical home” model in which care is comprehensive, coordinated, and provided to individuals with developmental disabilities and their families across the life course regardless of chronological age [50].

### 4.3. Gaps in Services

Caregivers identified numerous gaps in the current service landscape, one of which was in transition planning. Although federal law requires that all special education students receive transition planning, only 58% of youth with ASD receive those services by the required age [1]. Some best practices to support families during the transition process have been identified, including education, empowerment, and participation in planning [51]. Caring for youth was alternately identified by caregivers as a source of conflict and cohesion in the family, suggesting that additional family support is also needed. Support groups for parents and siblings are among the top family-requested services in families with school-age children with ASD [44]. As families provide the foundation of long-term care for those with ASD, it is imperative to adequately support them. Yet, a recent systematic review of interventions to mitigate stress and anxiety among caregivers of ASD youth identified only two studies, out of the 13 available, that focused specifically on caregivers of transition-age youth [52]. Furthermore, while numerous interventions exist that train typically developing siblings as therapy agents (e.g., siblings teach, model, or prompt) to improve ASD youth's social or behavioral skills, far fewer interventions have been developed to address siblings' psychosocial needs [53]. Lifelong, family systems-based interventions focused on sibling support are critical given the complexities of families affected by ASD as well as siblings' influential role in caring for ASD youth through adulthood [54, 55].

The need for crisis stabilization services has received little attention in prior research. Several families in the current study lamented the lack of options available to safely manage serious behavioral and mental health symptoms displayed by youth with ASD. This finding comports with those of White et al. [56], who found that families experience crises as pervasive and feel alone in trying to address them. Providers could assist families by organizing short-term residential crisis response services modeled, for example, after successful efforts in Dane County, Wisconsin [57].

The need to enhance existing vocational training and employment support for those with ASD has been documented in prior quantitative studies in the U.S. [45] and internationally [17, 58]. Our caregivers requested a greater focus on vocational skills and provision of work opportunities, especially in preparing youth for competitive employment. Caregivers also recognized the interrelatedness of social skills and employment among youth by requesting more social skills training and opportunities to develop daily living skills transferable to “real life.” Recent work demonstrates the feasibility of targeting ASD-specific social skills deficits within vocational rehabilitation programs [20].

Beyond the need for specific services, caregivers expressed the necessity of age-appropriate ASD programming for individuals with diverse levels of functioning. The struggle to receive services appropriately tailored to individuals on diverse points of the autism spectrum, as well as those with comorbid mental and physical conditions, has been reported by parents of young children [59] and young adults with ASD [18]. Our caregivers also identified a need for greater inclusivity for racial and ethnic minority families and sexual or gender minority ASD youth. Caregivers urged providers to prepare for cohorts of emerging adults with increasingly diverse clinical and demographic profiles, and such intersectionality will be an important topic to address in future research.

### 4.4. Education

Caregivers in this study directed service providers to improve education about ASD at all levels, including for direct care staff, educators, medical doctors, families, and general society. The need for increased training and education of direct care staff has been identified in some prior studies of specific programs [19] or populations (e.g., foster care youth with ASD [43]). Consistent with some past research [18], caregivers in the current study expressed frustration at having to educate service providers about ASD. Indeed, research suggests that, internationally, in-depth training on ASD and evidence-based practices to support those with ASD are severely lacking for health, education, and social care providers [60] and that ASD stigma endures via multiple myths held by the lay public [61]. Efforts to enhance provider training and increase societal awareness of ASD could help reduce feelings of burden and isolation among family caregivers.

### 4.5. Build Relationships

From the perspective of caregivers, it is crucial that providers build rapport and relationships with families and youth with ASD in order to effectively deliver services. This dovetails with the findings of other studies indicating, for example, that adults with ASD describe a lack of mutual understanding, communication, and trust with medical professionals as factors rendering primary care physical exams difficult [62]. Our study underscores listening as a critical skill in relationship-building, echoing previous work in which parents of children and adolescents with ASD feel that healthcare professionals do not listen to them or integrate them into decision-making [22, 39]. These factors are important since the quality of the parent-provider relationship has been found to impact perceptions of ASD service adequacy [63].

In addition to rapport-building, caregivers in our study advised intentional collaboration with families, drawing on families' expertise in caring for their youth with ASD. This aligns with Smith and Anderson's [64] call to incorporate “family-centered transition programming into school and clinical settings” (p. 118). Integrated as a whole, this study's findings suggest a need for holistic, family-centered approaches to care that target both provider-family relationships as well as training and services for autistic youth and support for family units. Prior work notes that lifelong models of parent and caregiver training are aspirational [65], but that very little research has examined optimal caregiver interventions for adults with autism [66]. In building family-centered approaches to care, effort should be taken to minimize potential incongruence between how such care is enacted by providers and how systems operate [37]. Future inquiry should explore the extent to which such perceived incongruence impacts family-provider relationship quality.

### 4.6. Policy Implications

Compared to other states, Ohio is considered average in terms of ASD-mandated coverage for private insurers [67], eligibility requirements, coverage, and spending limits under Medicaid and Medicaid waiver programs [68, 69], and the overall availability of autism services in the community [70]. As such, the study's findings suggest numerous policy implications. First, despite the well-documented increase in reported ASD prevalence in recent decades, very few states, Ohio included, offer Medicaid waivers that are specific to ASD [71] and even fewer offer ASD-specific waivers for adults [72]. Designing ASD-specific waivers may better address caregivers' identified needs for increased availability and better access to specialized services most relevant to transition-age individuals with ASD. While research on the effectiveness of ASD-specific waivers in improving youth and family outcomes is still developing, early findings suggest benefits for both ASD youth (e.g., improved independent living skills) and families' quality of life [73].

Though not specific to autism diagnoses, recent child services advocacy efforts in Ohio have led to the development of a new state Medicaid waiver (1915c-OhioRISE) [74] that provides additional support for multisystemic children and youth up to 20 years of age who have significant behavioral health treatment needs. Dovetailing with caregivers' advice in this study to improve service access and better understand and address youths' behavioral symptoms, caregivers of transition-age youth through 20 years old in Ohio may leverage services from this waiver. Relatedly, autism-specific advocacy efforts in the state are currently seeking to enhance applied behavioral analysis (ABA) services, and legislation is presently being drafted to require the Ohio Department of Medicaid to collect data on ABA therapy for enrolled youth diagnosed with ASD to better document service access, use, and unmet need [68]. Future policy initiatives should make use of existing advocacy coalitions driving these service expansions to build data for ongoing evidence-based program improvements.

Study findings additionally underscore the need for more affordable quality services, as some caregivers noted financial strain and the limited pool of providers who accept Medicaid insurance. Medicaid payment rates are known to be substantially lower than those of private insurance [75, 76]. Moreover, research evidences that providers additionally encounter more billing problems with Medicaid compared to private insurers and Medicare, generating an administrative burden that de-incentivizes providers to accept Medicaid clients. Policy decisions that increase state budgets and streamline Medicaid payment processes are likely to improve access to quality services [77].

## 5. Conclusion

### 5.1. Strengths and Limitations

This study was a small qualitative component of a broader study. Thus, the collection of expansive qualitative data was not possible. Our study was conducted in one U.S. state, and while 14 counties were represented, most caregivers were from one large metropolitan county. Although participants' experiences are certainly influenced by how services are funded and structured locally, our findings align with those from other parts of the U.S. and internationally. For instance, parents in Canada perceive the care system as overly complicated [40] and caregivers in both the U.K. [36] and Australia [78] express frustration with having to “fight” for and coordinate services. It is possible that our data miss service system-specific nuances due to the general nature of the interview questions. However, it is important to note that participants' relationship-building advice applies to providers within all service systems. Future research might explore how caregiver advice may differ, qualitatively and quantitatively, across specific systems, as well as the extent to which youth characteristics (e.g., age, racial and ethnic identity, functioning) predict variation in caregiver perceptions.

Our sample included a smaller proportion of non-white minoritized and socioeconomically marginalized families as compared to the whole U.S. Consistent with the experiences of many other researchers, our recruitment efforts did not effectively reach many minoritized and low-income families. This may partially reflect failures of service-delivery systems, which hinder effective service use by minoritized and low-income families [79], as well as families headed by caregivers with lower levels of education [80, 81]. Notably, however, our findings suggest that the experience of caring for minoritized youth with ASD may differ in ways that affect their safety and well-being.

Despite limitations, our study includes multiple strengths. First, we explicitly solicited family caregivers' advice about how providers could improve services for youth with ASD. Our study expands on a small body of prior research that has collected caregiver perspectives on distinct experiences such as the transition from high school [15, 18] or the receipt of specific services such as vocational rehabilitation [20], recreation [21], or primary care [22]. Second, unlike prior studies that have included other stakeholders such as youth with ASD, program staff, and policymakers [15, 19], the current study focused solely on the recommendations of family caregivers. Thus, we were able to identify unique needs (e.g., roadmapping, sibling supports, crisis care) and perspectives (e.g., burden of care coordination assumed by families, strategies for enhancing trust) not evident in prior work. Finally, our study can help provide a context from which to interpret data regarding satisfaction with services and unmet service needs. By identifying how care providers can better serve adolescents and young adults with ASD, participants in our study provide a critical voice to the many caregivers who are navigating the challenges associated with moving youth into adult systems of care.

## Figures and Tables

**Table 1:** Characteristics of family caregivers and youth with ASD (*N* = 174).

	*n* or *M*	% or (SD)	Range
*Family Caregiver Characteristics*			
Age, *M (*SD)	54.2	(6.8)	35–72
Female	159	91.4%	
Race/Ethnicity			
White	139	79.9%	
Black	26	14.9%	
Other (Hispanic, multiracial, or other)	9	5.2%	
Married	122	70.1%	
Employed (full-time or part-time)	120	69.0%	
4-year college degree or higher	105	60.4%	
Annual household income			
<$40,000	33	18.9%	
$40,000 to $74,999	42	24.1%	
$75,000 to $99,999	22	12.6%	
$100,000 to $150,000	40	23.0%	
$150,000 or more	37	21.3%	
Lives in large, central urban county	120	69.0%	

*Characteristics of Youth with ASD*			
Age, *M (*SD)	20.9	(3.7)	16–30
Male	125	71.8%	
Lives with caregiver	142	81.6%	
Primary diagnosis			
Autism	119	68.4%	
Asperger's	27	15.5%	
Other (other, multiple, or unknown)	28	16.1%	
Comorbid mental health disorder	106	60.9%	
Comorbid intellectual disability	47	27.0%	
School and work status			
In high/secondary school	71	40.8%	
In college or vocational school	22	12.6%	
Out of school, working full-time or part-time	49	28.2%	
Out of school, not working	32	18.4%	
Level of support needed^*a*^			
None or infrequent	30	17.2%	
Intermittent	67	38.5%	
Limited, but consistent	32	18.4%	
Frequent and close	28	16.1%	
Extensive or continuous	17	9.8%	
Has Medicaid coverage	100	57.5%	
Has Medicaid waiver for services	59	33.9%	

^*a*^index based on the scales for independent living-revised short form [25].

## Data Availability

The interview data used to support the findings of this study have not been made available because participants did not consent to their individual confidential data being shared publicly. Kristen A. Berg and Karen J. Ishler had full access to all of the data in the study and take responsibility for the integrity of the data and accuracy of the analysis.
